# Single cell RNA-sequencing in uveal melanoma: advances in heterogeneity, tumor microenvironment and immunotherapy

**DOI:** 10.3389/fimmu.2024.1427348

**Published:** 2024-06-20

**Authors:** Shiyi Tang, Yun Zhang, Shengmei Huang, Tengfei Zhu, Xiaojing Huang

**Affiliations:** ^1^ Department of Ophthalmology, Shanghai Pudong New Area Gongli Hospital, Shanghai, China; ^2^ Department of Ophthalmology, Changzheng Hospital, Second Affiliated Hospital of Naval Medical University, Shanghai, China; ^3^ Department of Anesthesiology, Changzheng Hospital, Second Affiliated Hospital of Naval Medical University, Shanghai, China

**Keywords:** uveal melanoma, single-cell sequencing, tumor microenvironment, immunotherapy, prognostic model

## Abstract

Uveal melanoma (UM) is a highly aggressive and fatal tumor in the eye, and due the special biology of UM, immunotherapy showed little effect in UM patients. To improve the efficacy of immunotherapy for UM patients is of great clinical importance. Single-cell RNA sequencing(scRNA-seq) provides a critical perspective for deciphering the complexity of intratumor heterogeneity and tumor microenvironment(TME). Combing the bioinformatics analysis, scRNA-seq could help to find prognosis-related molecular indicators, develop new therapeutic targets especially for immunotherapy, and finally to guide the clinical treatment options.

## Introduction

Uveal melanoma (UM) is the most common primary intraocular malignancy in adults, accounting for 3%-5% of all melanoma (1). UM originates from melanocytes of the iris (3–5%), ciliary body (5–8%), or choroid (approximately 85%) (2). The average incidence of UM in the world is 0.0001%~0.0009%, with obvious regional and ethnic differences, with the highest incidence in white people, followed by yellow people, and less common in black people (3). The incidence is slightly higher in males than in females. The advances of diagnosis and treatment have improved the local control rate of UM, but the overall survival(OS) remains unchanged (4). About 50% of patients with UM eventually develop metastases, most involving the liver (5). And the median OS for metastatic UM(mUM) patients is shorter than 12 months (6). Improving the survival of UM patient is the main clinical goal at present.

Single-cell RNA sequencing(scRNA-seq) is a cutting-edge technology that could provide in-depth information of tumor heterogeneity and tumor microenvironment(TME) (7, 8) and has yielded remarkable results towards the molecular biology of UM. Combined with bioinformatic analysis, scRNA-seq could provide detailed information regarding the occurrence, progression and metastasis of UM and help to develop new therapeutic targets and prognostic models (9). Immunotherapy has been widely used in anti-tumor therapy, but UM shows little response to immunotherapy as it has the lowest tumor mutation burden in all types of cancer (10, 11). Moreover, eyes are immune privileged and could protects the eye tissue from the immune system attack (12). As is known that the efficacy of immunotherapy is closely related the TME (13), using scRNA-seq to analyze the features of TME in UM patients could give insight to improve the efficiency of immunotherapy for UM patients. In this review, we are going to summarize the recent progresses on the application of scRNA-seq in UM and explore their insight to immunotherapy (**Table 1**).

**Table 1:** Overview of application of scRNA-seq in UM.

1.Tumor heterogeneity		
Samples	Key findings	Reference
Primary and metastatic UM with or without BAP1 mutations (MUTs) from GEO database(GSE138433, GSE139829 and GSE176029)	• Identified Cluster 1 tumor cells expressed high levels of genes linked to tumor metastasis. • communication between terminally exhausted CD8+ T cells and GDF15^hi^ATF3^hi^CDKN1A^hi^ tumor cells was enhanced in BAP1-mutated UM and CellChat analysis predicting strong ITGB2-ICAM1 signaling between • The inhibitors that inhibited hypoxia- and ECM-related pathways could prevent liver metastasis in the BAP1-mutated group	(16)
Six different primary UMs	• uncovered an intratumoral heterogeneity at the genomic and transcriptomic level. • deciphered a gene regulatory network underlying an invasive and poor prognosis state driven in part by the transcription factor HES6 • HES6 is a valid target to stop uveal melanoma progression	(17)
TCGA and GSE22138 cohorts	• Divided UM into two different immune subtypes • Different subsets were involved in different TF-Target gene regulatory networks.	(18)
Liver metastases from a patient with mUM	• Identified intra- and intertumoral heterogeneity of the two metastases, indicating the complexity of heterogeneity in UM	(19)
Six freshly enucleated UM specimens from different prognostic categories	• Polycomb repressor complex 1(PRC1) was a key step in UM progression • The deregulation of PRC1 can promote tumor progression by inducing chromosomal instability, and targeting chromosomal instability could be an effective strategy of early therapeutic intervention in UM	(20)
2.Biomarkers and predictive models		
Samples	Types of predictive models	Reference
GSE139829 and TCGA	Necroptosis-related prognostic model	(26)
GSE22138, GSE44295, GSE84976 and TCGA	Immune-related gene signature for prognosis	(27)
GSE84976 and TCGA	Lipid metabolism-related risk model	(28)
GSE139829 and TCGA	(m7G)-related prognostic signature	(29)
GSE84976, GSE22138 and TCGA	Basement membrane genes related prognostic signature	(30)
GSE139829, GSE84976 and TCGA	Glycosylation-based gene signature for prognostic prediction	(31)
GSE139829, GSE84976, GSE22138 and TCGA	Hypoxia-related nine-gene prognostic model	(32)
GSE135922 and TCGA	FOXD1 exclusively expressed in high-risk UM and its expression is associated with a poor prognosis	(33)
3.TME and insight into immunotherapy		
Samples	Key findings	Reference
Eight primary and three metastatic Ums	• Identified a previously unrecognized subtype of CD8+ T cells that predominantly expressed LAG3 • LAG-3 would be a potential target in immune checkpoint inhibitors in UM patients	(40)
GSE84976 and TCGA	• MGLL was strongly expressed in M2 macrophages • MGLL might be a potential target for the immunotherapy of UM patients	(28)
GSE138433, GSE139829 and GSE176029	• Terminally exhausted CD8+ T cells and GDF15^hi^ATF3^hi^CDKN1A^hi^ tumor cells was enhanced in BAP1-mutated UM and CellChat analysis predicting strong ITGB2-ICAM1 signaling between	(16)
GSE84976, GSE22138 and TCGA	• ITGA5 showed predominant expression in CD8 +Tex and CD8+ T immune cell populations	(30)
GSE139829 and TCGA	• NQO1 mRNA expression levels and the infiltration of immune cells and stromal cells	(43)
CancerSEA database	• NT5E was associated positively with the infiltration of cancer-associated fibroblasts	(44)

## Tumor heterogeneity

UM a highly heterogeneous and complex disease, and intertumoral heterogeneity has been observed with markedly different genetic backgrounds, histopathological features, and clinical behaviors. Apart from intertumoral heterogeneity, intratumoral heterogeneity (ITH) has only been recognized within the past decades with the advances in sequencing technology. ITH originates from the genetic heterogeneity of cancer cells and could lead to distinct populations present in the tumor (14, 15). Each clone has unique functional properties, such as the ability to form metastases or respond to specific therapies.

BAP1 inactivating mutations was a risk factor for metastasis in UM patients. To understand the mechanisms of BAP1 deficiency driving UM metastasis, Jiaoduan Li et al. analyzed scRNA-seq data that included primary and metastatic UM with or without BAP1 mutations (MUTs). The malignant cells were divided into five clusters, and malignant cells expressing high levels of GDF15, ATF3, and CDKN1A were linked to tumor metastasis (16). Charlotte Pandiani et al. use single-cell RNA sequencing of six different primary uveal melanomas and uncovered distinct intratumoral heterogeneity at the genomic and transcriptomic level. Further analysis identified a gene regulatory network underlying an invasive and poor prognosis state driven by the transcription factor HES6 (17). By integration of bulk RNA-seq and scRNA-seq, Guohong Gao et al. had divided UM patients into two different immune subtypes and each subtypes a had its unique characteristics in prognosis, immune-related molecules, immune score, and immune cell infiltration (18). Weitao Lin et al. utilized scRNA-seq of two metastases from a mUM patients who had received immunotherapy and the results demonstrated that two metastases were largely infiltrated by noncancerous cells with significant variability in cell types. Further single-nucleotide polymorphism(SNP) and copy-number variations (CNVs) uncovered both intra- and intertumoral heterogeneity of the two metastases, indicating the complexity of heterogeneity in UM (19). Mathieu F. Bakhoum et al. explored the relationship between chromosomal instability, epigenetics, tumor progression and metastasis by integrating information by scRNA-seq, and the results revealed that alterations in a series of molecules driven by the deletion of Polycomb repressor complex 1(PRC1) was a key step in UM progression. The deregulation of PRC1 can promote tumor progression by inducing chromosomal instability, and targeting chromosomal instability could be an effective strategy of early therapeutic intervention in UM (20).

These results above verified the existence of ITH within the same tumor, and each subset of heterogeneity had its unique characteristics. ScRNA-seq allows the researchers to evaluate the genetic heterogeneity at single-cell resolution and helps to reveal the complex biological diversity within the tumor and to make the individualized treatment plan.

## Biomarkers and predictive models

Based on the 8^th^ edition AJCC cancer staging, UM is classified by tumor size (diameter and thickness), anatomical extent (ciliary involvement, and extrascleral extension) (21). With the advances of genetics, the deletion of chromosome 3 and the increase of chromosome 8 have been found to be associated with poor prognosis (22). Therefore, Dogrusöz et al. and Bagger et al. added chromosomal information to the AJCC staging system to further improve the prognostic prediction (23). In 2004, Onken et al. used unsupervised clustering in primary UM transcriptome analysis and uncovered two GEP-based classifications, one with a better prognosis and the other with a poor prognosis (24). With the application of next-generation sequencing technology, the driver genes were discovered in UM development. The primary driver genes are GNAQ, GNA11, and secondary driver genes are BAP1, SF3B1, EIF1MX (25). About 90% of UM patients carry GNAQ and GNA11 gene mutations, which activate the tumorigenesis. BAP1 mutations have the worst clinical prognosis, followed by SF3B1 and EIF1MX (25). ScRNA-seq could provide in-depth information about mechanism of tumorigenesis and tumor progression and could avoid the ITH with the tumor brought by the bulk RNA-seq. Biomarkers and prediction model based on the scRNA-seq data might be more accurate than those based on the bulk RNA-seq.

Jiaheng Xie et al. used the online scRNA-seq data of UM, and identified a hub of necroptosis-related genes. By COX regression and Lasso regression, the authors constructed a necroptosis-related prognostic model that could distinguish different survival time (26). Wanpeng Wang et al. identified the immune infiltration pattern of UM and constructed an immune-related gene prognostic signature which had strong predictive ability for UM patients (27). Similarly, Yao Tan et al. investigated the expression patterns of lipid metabolism in UM patients and established a risk model based on the genes involved with lipid metabolism which could accurately predict survival in patients with UM (28). Moreover, researchers had constructed a m7G-related prognostic signature by integrating TCGA and GEO database and suggested PAG1 as biomarker for diagnosis and treatment of UM (29). Yunyue Li et al. also developed a prognostic risk model based on basement membrane protein-related genes (30), and glycosylation-based gene signature and hypoxia-related gene signature were described as well by integrating single-cell analysis and machine learning (31, 32). Re-analyzing publicly available single cell RNA sequencing, researchers found that FOXD1 exclusively expressed in high-risk UM and its expression is associated with a poor prognosis, suggesting FOXD1 as a new biomarker for the diagnosis of UM (33).

Base on the scRNA-seq data, especially those online public data, researchers have developed a series of prediction model and biomarkers with the intention to accurately predict the prognosis of UM patients. However, the clinical validation of those models and biomarkers is still uncertain. Apart from developing new biomarkers and models, the validation seems more practical.

## TME and insight into immunotherapy

Although UM originated from melanocytes, the biological and clinical features of UM are quite different from cutaneous melanoma(CM). CM harbors higher tumor mutation burdens(TMB) and shows good response to immune checkpoint inhibitos(ICIs) (34). High TMB could lead to the continuous production of neoantigens which makes the tumors highly immunogenic and sensitive to ICIs (35). However, UM has the lowest TMB among all types of tumors, which makes it irresponsible to ICIs (36). Besides the TMB, the response to ICIs is closely related to TME. TME is mainly composed of tumor cells and surrounding tumor-associated immune cells, such as tumor-infiltrating lymphocytes (TILs), natural killer (NK) cells, tumor-associated non-immune cells (fibroblasts, lipid cells, etc.), and extracellular matrix(ECM), which plays a key role in the occurrence, progression, and metastasis of tumor. As previously described, constant interactions between tumor cells and their surrounding microenvironment was closely related with treatment response and prognosis of malignancies (37, 38). ScRNA-seq is a powerful tool to explore the complex TME, and exploring the mechanism of the interaction between TME and tumor cells may lead to a breakthrough in immunotherapy for UM.

Michael A Durante et al. interrogated the tumor microenvironment using scRNA-seq of 59,915 tumor and non-neoplastic cells from 8 primary and 3 metastatic samples. The tumor-infiltrating immune cells comprised a previously unrecognized subtype of CD8+ T cells that predominantly expressed the checkpoint marker LAG3, rather than PD-1 and CTLA-4, indicating that LAG-3 would be a potential target in immune checkpoint inhibitors in UM patients (39). LAG-3, like PD-1 and CTLA-4, is another ICI protein. LAG-3 is not expressed on naïve T cells, but can be induced on CD4+ and CD8+ T cells when stimulated by antigens to inhibit T cell function (40). It has been reported that anti-PD-1 and anti-CTLA-4 cannot improve the prognosis of mUM patients (41), and anti-LAG-3 might be a new direction to the immunotherapy of UM patients. Yao Tan et al. explored the expression patterns of lipid metabolism in 80 UM patients from the TCGA database and integrated the results with single-cell sequencing analysis on UM patients from the GEO data to characterize the lipid metabolism in TME. And the results showed that monoacylglycerol lipase (MGLL) was strongly expressed in macrophages, specifically M2 macrophages, which might function in the M2 polarization and M2 macrophage activation, suggesting that MGLL might be a potential target for the immunotherapy of UM patients (28). Macrophages that recruited in TME are mostly M2 phenotype, which plays a critical role in promoting tumor progression (42). Strategies targeting tumor-associated macrophages(TAMs) are reducing the number of TAMs or altering their functionality within the TME. And by uncovering the specific markers on TAMs, targeted antibodies towards those markers could be applied to either eliminate or disfunction those tumor -promoting TAMs. In BAP1-mutated UM, researchers found that ITGB2-ICAM1 signaling was enhanced between terminally exhausted CD8+ T cells and GDF15^hi^ATF3^hi^CDKN1A^hi^ tumor cells, and inhibiting either ICAM1 or ITGB2 could prevent liver metastasis in the BAP1-mutated patients (16). In the study of Yunyue Li et al, ADAMTS10 and ITGA5 were expressed in various immune cell, and ITGA5 showed predominant expression in CD8 +Tex and CD8+ T immune cell populations. Further studies should be carried out to study the function of those basement membrane protein-related genes in TME and immunotherapy of UM (30). Liping Shen et al. conducted a pan cancer research of NQO1 and found that NQO1 was significantly upregulated in most cancer types. The scRNA-seq analysis revealed a potential relationship between the NQO1 mRNA expression levels and the infiltration of immune cells and stromal cells, and inhibition of NQO1 might be a promising strategy for cancer immunotherapy (43). Likewise, Xinmiao Xue et al. identified NT5E as a novel prognostic biomarker in a pan-cancer analysis. The overexpression of NT5E was related to worse overall survival in UM patients and NT5E was associated positively with the infiltration of cancer-associated fibroblasts (CAFs) through epithelial-mesenchymal transition(EMT) which provided the insight for immunotherapy by targeting EMT (44).

The existing anti-PD-1/PD-L1 and anti-CTLA-1 antibodies showed little response in UM patients. Although studies have demonstrated that LAG-3 might be a promising ICIs in UM, little is reported about the clinical application anti-LAG-3 antibody in UM patients. With the improvements of scRNA-seq, detailed analysis of TME could be carried out and more ICIs and other targeted therapies would be developed to improve the immunotherapy of UM. 

## Discussion

ScRNA-seq is promising technology that could provide thorough information of a single cell and could also address the complex TME. The adoption of scRNA-seq has provided us the deeper understanding of the gene expression, and cell type, sub-species type, state and development trajectory within the tumor. But there are still some limitations about scRNA-seq. As to apply the scRNA-seq, tissues must to be digested into single cells. During the digestion cell integrity and cell viability are often compromised and the spatial location of tumor cells is also lost which is a very important information in TME. The recently developed spatial transcriptomics(ST) could provide the gene expression profile within intact tissue (45), which would be perfect supplement to scRNA-seq. And by integrating ST and scRNA-seq should be more valuable to study the molecular mechanisms and TME. Another limitation is the high cost of scRNA-seq, which have greatly limited its application across the world. Reduced sequencing costs would facilitate the widespread use of scRNA-seq in tumor research.

As the most common primary intraocular malignancy in adults, UM patients can seldom benefit from immunotherapy due to the special immune characteristics of UM- low tumor mutation burden and low immune infiltration. Although there were no major breakthroughs in the immunotherapy in UM, the immune system still plays an important role in the development of UM. With the adoption of scRNA-seq in tumor research, hopes have been lit up upon the immunotherapy in UM patients. Traditional bulk RNA-seq is difficult to capture the heterogeneity within the tumor, but scRNA-seq could provide detailed information about the interaction between tumor cells and immune cells in TME, which would be the potential target for immunotherapy (46). New immune check point proteins and molecular mechanisms have been uncovered by illustrating the comprehensive picture of TME in UM by scRNA-seq. And clinical validations should be carried out to confirm the findings obtained in scRNA-seq. However, the overall results are still insufficient and a quite number of studies used the online public data. More detailed studies should be encouraged by scRNA-seq. With the development of scRNA-seq, more in-depth mechanisms of UM have been uncovered and by designing the specific drugs targeting the mechanisms, the treatment would be more effective and thus prolong the survival of UM patients.

## Author contributions

ST: Conceptualization, Writing – original draft. YZ: Formal analysis, Investigation, Writing – review & editing. SH: Investigation, Writing – review & editing. TZ: Investigation, Writing – review & editing. XH: Conceptualization, Funding acquisition, Investigation, Writing – review & editing.
